# Update on the urotensinergic system: new trends in receptor localization, activation, and drug design

**DOI:** 10.3389/fendo.2012.00174

**Published:** 2013-01-02

**Authors:** David Chatenet, Thi-Tuyet M. Nguyen, Myriam Létourneau, Alain Fournier

**Affiliations:** ^1^Laboratoire d'études moléculaires et pharmacologiques des peptides, INRS – Institut Armand-Frappier, Université du Québec, Ville de LavalQC, Canada; ^2^Laboratoire International Associé Samuel de Champlain (INSERM/INRS-Université de Rouen)France

**Keywords:** urotensin II, urotensin II-related peptide, allosteric modulation, biased agonist, nuclear receptors

## Abstract

The urotensinergic system plays central roles in the physiological regulation of major mammalian organ systems, including the cardiovascular system. As a matter of fact, this system has been linked to numerous pathophysiological states including atherosclerosis, heart failure, hypertension, diabetes as well as psychological, and neurological disorders. The delineation of the (patho)physiological roles of the urotensinergic system has been hampered by the absence of potent and selective antagonists for the urotensin II-receptor (UT). Thus, a more precise definition of the molecular functioning of the urotensinergic system, in normal conditions as well as in a pathological state is still critically needed. The recent discovery of nuclear UT within cardiomyocytes has highlighted the cellular complexity of this system and suggested that UT-associated biological responses are not only initiated at the cell surface but may result from the integration of extracellular and intracellular signaling pathways. Thus, such nuclear-localized receptors, regulating distinct signaling pathways, may represent new therapeutic targets. With the recent observation that urotensin II (UII) and urotensin II-related peptide (URP) exert different biological effects and the postulate that they could also have distinct pathophysiological roles in hypertension, it appears crucial to reassess the recognition process involving UII and URP with UT, and to push forward the development of new analogs of the UT system aimed at discriminating UII- and URP-mediated biological activities. The recent development of such compounds, *i.e.* urocontrin A and rUII(1–7), is certainly useful to decipher the specific roles of UII and URP *in vitro* and *in vivo*. Altogether, these studies, which provide important information regarding the pharmacology of the urotensinergic system and the conformational requirements for binding and activation, will ultimately lead to the development of potent and selective drugs.

## The urotensinergic system

**Figure 1:**
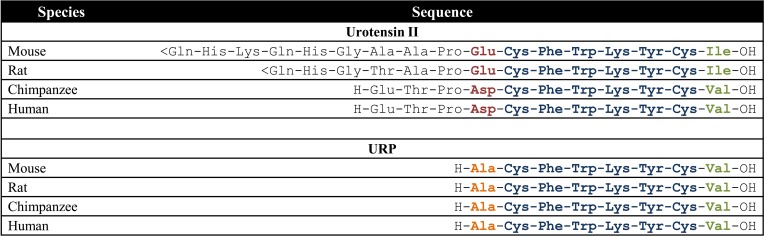
**Amino acid sequences of UII and URP in mammalian species; <Gln, pyroglumatic acid.** Modified from Vaudry et al. (2010).

**Figure 2:**
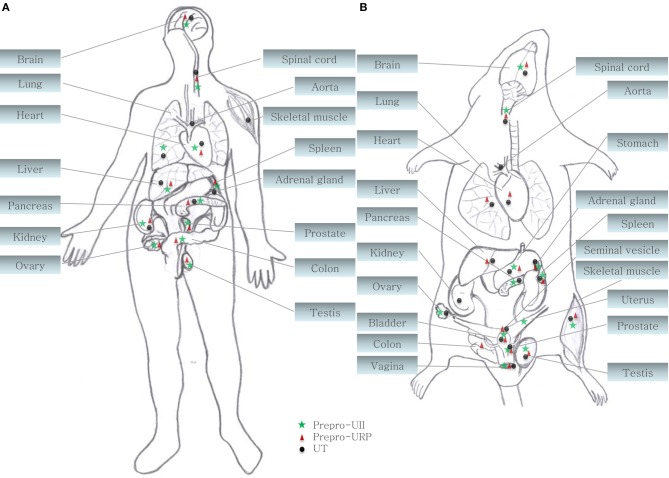
**Central and peripheral distribution of prepro-UII, prepro-URP, and UT in **(A)** primates (human and cynomolgus monkey) and **(B)** rodents (rat and mouse) (Ames et al., 1999; Sugo et al., 2003; Dubessy et al., 2008; Maguire et al., 2008; Doan et al., 2012; Nguyen et al., 2012)**.

During the last decade, the urotensinergic system has drawn the attention of the scientific community due to its marked involvement in various pathological states including cardiovascular diseases. Initially isolated from the caudal neurosecretory system of the teleostean fish *Gillichthys mirabilis*, urotensin II (UII), a somatostatin-like peptide, was first characterized as a spasmogenic agent (Pearson et al., 1980). During more than 15 years, this peptide and its unknown receptors were thought to be restricted to fishes until it was demonstrated that UII was able to induce the relaxation of the mouse anococcygeus muscle (Gibson et al., 1984) and provoke the contraction of rat aortic strips (Gibson, 1987). These results, suggesting the presence of an homologous peptide in higher vertebrates, led to the isolation and characterization of UII in the frog *Rana ridibunda* (Conlon et al., 1992). Following this discovery, UII isoforms were either characterized or isolated in various vertebrate species including humans (Vaudry et al., 2010). A few years later, a peptide paralog, termed urotensin II-related peptide (URP), was isolated in rat brain extracts and subsequently identified in other mammalian species (Vaudry et al., 2010). Sequence comparison of all UII and URP isoforms revealed a striking conservation of the C-terminal cyclic hexapeptide (Vaudry et al., 2010). Conversely, the N-terminal region is highly variable both in length, ranging from 11 residues in humans to 17 residues in mice, and sequence composition (Figure 1) (Vaudry et al., 2010). In the human genome, UII and URP genes are respectively found at position 1p36 and 3q29 (Sugo et al., 2003). Those two genes are primarily expressed in motoneurons located in discrete brainstem nuclei and in the ventral horn of the spinal cord (Vaudry et al., 2010). However, UII and URP mRNAs have also been detected, although at a much lower level, in various peripheral tissues including the pituitary, heart, spleen, lung, liver, thymus, pancreas, kidney, small intestine, adrenal, and prostate (Figure 2) (Vaudry et al., 2010).

**Figure 3:**
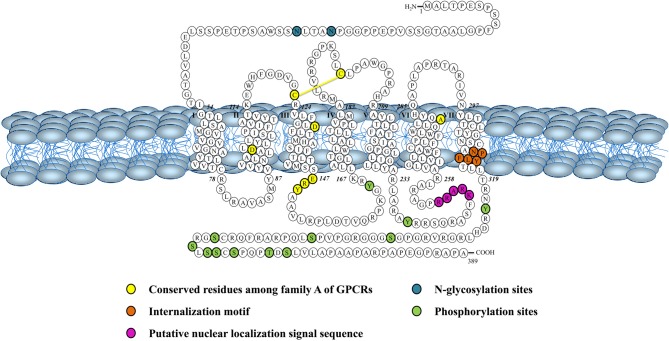
**Schematic representation of the human urotensin II (hUT) receptor.** Adapted from Douglas et al. (2000).

Both peptides are endogenous ligands of a G protein-coupled receptor initially identified as the orphan GPR14 receptor (Ames et al., 1999; Liu et al., 1999; Mori et al., 1999; Nothacker et al., 1999). Structural studies of this urotensin II receptor (UT) showed that, in addition to the common features found in the 1A GPCR family, such as the existence of a disulfide bridge between extracellular loops 1 and 2, N-linked glycosylation sites in the N-terminus portion, and phosphorylation sites in intracellular loops (Douglas et al., 2000), this protein also possesses a palmitoylation site located in the C-terminal segment of the rodent isoform that is not present in the human isoform (Figure 3). Worth to mention, the rat UT, consisting of 386 amino acids, shows only 75% homology with the human protein while sequences of human and monkey receptors, comprising 389 residues, are almost identical (Elshourbagy et al., 2002). Like UII and URP, UT is widely expressed in the central nervous system as well as in various peripheral organs including the cardiovascular system, kidneys, bladder, pancreas, and adrenal gland (Figure 2) (Vaudry et al., 2010).

The urotensinergic system plays a seminal role in the physiological regulation of major mammalian organ systems, including the cardiovascular system (Vaudry et al., 2010). As a matter of fact, UII exerts potent haemodynamic effects (Krum and Kemp, 2007), positive inotropic and chronotropic responses (Watson et al., 2003), and osmoregulatory actions (Song et al., 2006), induces collagen and fibronectin accumulation (Dai et al., 2007; Zhang et al., 2008), modulates the inflammatory response (Shiraishi et al., 2008), plays a role in the induction of cardiac and vascular hypertrophy (Papadopoulos et al., 2008), causes a strong angiogenic effect (Guidolin et al., 2010) and inhibits the glucose-induced insulin release (Silvestre et al., 2004). Thus, the urotensinergic system was linked to numerous pathophysiological states including atherosclerosis, heart failure, hypertension, pre-eclampsia, diabetes, renal and liver diseases, variceal bleeding, ulcers, as well as psychological, and neurological disorders (Ross et al., 2010).

The present review focuses on the latest findings about the urotensinergic system in terms of receptor localization and pharmacology as well as receptor activation with the conception of new urotensinergic ligands aimed at discriminating UII- and/or URP-mediated biological actions.

## Discovery of an intracrine pharmacology of the urotensinergic system

### Presence of nuclear UT in the heart and in the central nervous system

**Figure 4:**
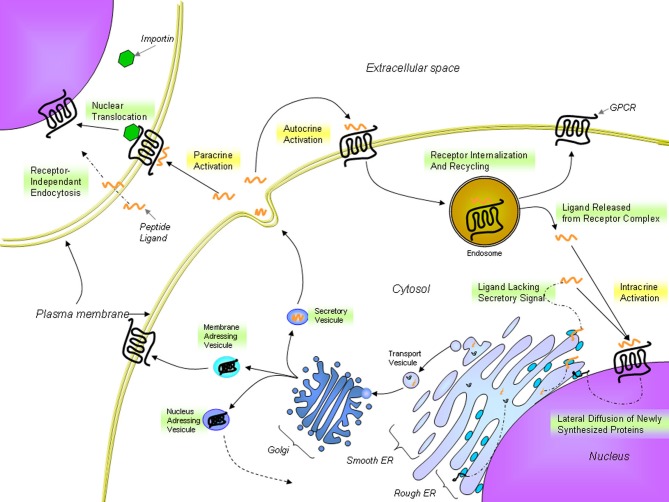
**Proposed trafficking of UT and endogenous ligands.** UT receptors are located either at the plasma membrane or at the nucleus. The presence of nuclear UTs can be attributed to lateral diffusion of newly synthesized proteins from the rough endoplasmic reticulum surface, which is contiguous with the nuclear shell, to specific nuclear addressing following modification in the Golgi apparatus or importin-assisted nuclear translocation after ligand stimulation. UII or URP binds to UT receptors at the cell surface but can also cross the plasma membrane to stimulate intracellular receptors. From Lee et al. (2004); Giebing et al. (2005); Gobeil et al. (2006); Doan et al. (2012); Tadevosyan et al. (2012).

In many ways, UII exhibits actions similar to other key neurohormonal factors, *i.e.* angiotensin II (Ang-II) and endothelin-1 (ET-1), in driving a variety of cardiac and vascular disease processes (Maguire and Davenport, 2002). These include vasoconstriction as well as mitogenic, trophic and pro-fibrotic effects (Vaudry et al., 2010). A clear interaction of the urotensinergic system with the renin-angiotensin-aldosterone and endothelin systems is acknowledged in terms of regulation of systolic and diastolic functions (Fontes-Sousa et al., 2009). However, key differences were observed between these systems. In particular, UII induces a rather weak or absent vasoconstriction in a variety of human vascular beds (Maguire et al., 2000; Hillier et al., 2001) while it can also acts as a vasodilator in some vascular beds, such as those in the pulmonary vasculature (Stirrat et al., 2001). The recent discovery of specific intracellular receptors associated with the physiological and pathophysiological actions of Ang-II and ET-1 highlighted a high level of complexity for these peptidergic systems in the regulation of cardiovascular homeostasis. Traditionally, GPCRs are located at the plasma membrane where they modulate the activity of membrane-associated second messengers. As such, GPCRs can exert their effects through the regulation of ion channels, second messenger production, and protein kinase cascades in order to control cellular activity, gene expression, plasticity, differentiation, morphogenesis, and migration. However, in the recent years, the presence of functional intracellular receptors has almost become “a classic GPCR paradigm” (Boivin et al., 2008). These intracellular GPCRs could be involved in the control of several cellular processes including regulation of gene transcription, ionic homeostasis, cellular proliferation, and remodeling (Boivin et al., 2008). Intracellular GPCRs may be constitutively active, or may be activated by ligands internalized from the extracellular space or synthesized within the cell (Figure 4). Besides, they can regulate signaling pathways distinct from those activated by the same receptor at the cell surface (Re, 1999). As such, biological outcomes might result from the integration of extracellular and intracellular signaling events (Terrillon and Bouvier, 2004; Hanyaloglu and von Zastrow, 2008; Sorkin and von Zastrow, 2009). This new paradigm for cellular signaling provides more complexity to study the function and physiological roles of GPCRs.

In a recent report, specific UII binding sites were observed on heart and brain cell nuclei from rat and monkey tissues (Doan et al., 2012; Nguyen et al., 2012). Except those two tissues and the spinal cord, none of the tested tissues including kidneys, lung, and skeletal muscle, all expressing UT at the cell surface, presented a subcellular localization of UT (Doan et al., 2012; Nguyen et al., 2012). Supporting the presence of such nuclear expression also in humans, the presence of nuclear UT was also observed in two human cell lines, *i.e.* SH-SY5Y neuroblastoma and U87 astrocytoma cell lines (Nguyen et al., 2012).

### Nuclear UT activation can modulate transcription initiation

As previously reported for nuclear Ang-II (Eggena et al., 1993), β3-adrenergic (Boivin et al., 2006; Vaniotis et al., 2011), and ET-1 receptors (Boivin et al., 2003), nuclear UT receptors can initiate transcription (Doan et al., 2012; Nguyen et al., 2012). Although UII and URP stimulated the transcription in isolated brain cell nuclei (Nguyen et al., 2012), only UII was able to trigger a similar effect in rat cardiac nuclei (Doan et al., 2012). Two-dimensional gel electrophoresis clearly indicated the occurrence of different immunoreactive species in both brain and heart membrane and nuclear fractions (Doan et al., 2012; Nguyen et al., 2012). Nuclear and membrane proteins extracted from heart tissues expressed three major UT-immunoreactive spots with an apparent molecular weight of 60 kDa at a pI value of 6–7 (Doan et al., 2012). Interestingly, a different pattern was observed in brain tissue (Nguyen et al., 2012). Since the UT gene is intronless, the various immunoreactive species were principally ascribed to post-translational modifications (Doan et al., 2012; Nguyen et al., 2012). Whether or not these UT species are involved in distinct UII-associated biological activities will require further investigation. However, it is well-known that glycosylation can modulate the cellular compartmentalization and functionality of the receptor, thereby influencing its intracellular trafficking and biological activity (Figure 4) (Duverger et al., 1995; Rondanino et al., 2003; Gobeil et al., 2006).

**Figure 5:**
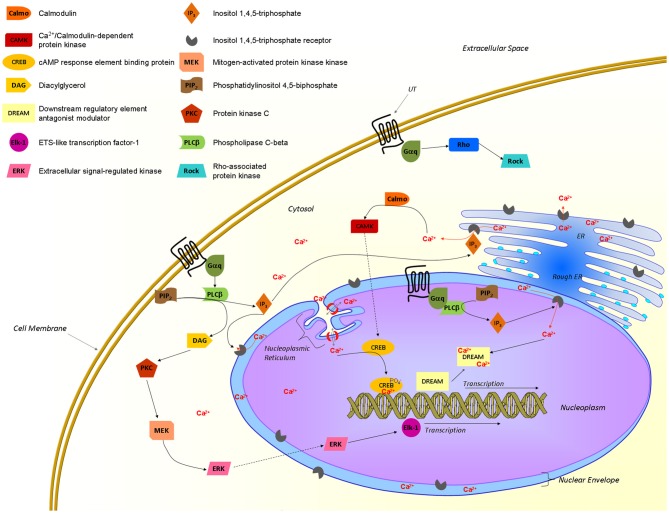
**Proposed UT-associated signaling pathways.** Activated UTs are known to recruit Gαq/11 proteins and to signal through different pathways associated with an elevation of intracellular calcium and gene transcription. Nuclear UTs could induce transcription through a pathway involving the production of IP_3_ that would trigger IP_3_Rs at the inner nuclear envelop, releasing calcium in the nucleoplasm. This latter event is crucial to initiate transcription through transcription factors such as CREB and DREAM. From Proulx et al. (2008); Bootman et al. (2009); Vaudry et al. (2010); Roskoski (2012); Tadevosyan et al. (2012).

A growing body of evidence supports the presence of GPCRs at the surface of the nuclear membrane, their orientation within this membrane, however, remains controversial. If they maintain the topology adopted in the endoplasmic reticulum during protein synthesis, the ligand binding site would be located in the lumen of the nuclear envelop (perinuclear space) with the C-terminal of the protein being localized either within or outside the nucleus. In fact, the topology of the nuclear membrane lumen is very similar to the extracellular space, which makes it a favorable environment for a binding site (Jong et al., 2005; Bootman et al., 2009). Since signaling starts with the recruitment of specific proteins to the C-terminal portion of the receptor, signals would be sent toward the cytosol or into the nucleus in accordance with the adopted GPCR orientation within the nuclear shell (Figure 5). Hence, the orientation of those nuclear GPCRs would determine the direction in which the signal is transmitted. As recently reported, nuclear UT receptors are able to regulate gene transcription (Doan et al., 2012; Nguyen et al., 2012). Furthermore, it is well-known that calcium ions play an important role in the control of gene expression (Bootman et al., 2009). In isolated nuclei, nuclear calcium levels can regulate gene transcription by interacting with the cyclic AMP response element-binding protein (CREB) and the downstream regulatory element antagonist modulator (DREAM), which are constitutively present in the nucleus. Changes in nucleoplasmic calcium can be achieved by triggering inositol(1,4,5)-triphosphate receptors (IP_3_Rs) located on the inner nuclear membrane (Bootman et al., 2009). Because it is generally accepted that UT activation is associated with the recruitment of Gαq/11 proteins to its C-terminal tail resulting in an IP_3_ increase (Proulx et al., 2008), it is highly probable that this portion of the receptor is located into the nucleoplasm (Figure 5). Interestingly, IP3Rs are concentrated in the nuclear membrane of heart ventricular cells and their activation was shown to initiate a pro-hypertrophic pathway (Arantes et al., 2012). These findings are well-correlated with the UII-induced cardiomyocyte hypertrophy and the presence of nuclear UT receptors in cardiac tissues (Gruson et al., 2010; Doan et al., 2012). Nevertheless, these intracellular UT receptors may have the capacity to regulate signaling pathways that differ from those of their plasma membrane counterparts, as recently demonstrated for the metabotropic glutamate receptor 5 (Jong et al., 2009), and the renin–angiotensin system (De Mello, 2008). As such, this intracrine pharmacology of the urotensinergic system represents a complementary system that could potentially involve the regulation of physiological functions.

### Intracellular trafficking of UT

This new intracrine pharmacology clearly highlights the complexity of this peptidergic system where UII and URP can trigger not only common but also different biological activities (Prosser et al., 2008; Jarry et al., 2010; Doan et al., 2012). Previous studies have detected the presence of GPCRs, such as Ang-II receptors, at the nucleus in an agonist-independent manner (Lee et al., 2004). Confocal microscopy of heart and brain tissue sections as well as various non-transfected cell lines clearly revealed a constitutive nuclear localization for UT (Doan et al., 2012; Nguyen et al., 2012). However, it is also possible that following their agonist-stimulated internalization, GPCRs relocate at the nuclear membrane (Lee et al., 2004). In such a case, the translocation is initiated by the presence of a nuclear localization signal (NLS), a short stretch of basic amino acid residues often localized within the intracellular loops that is recognized by importins α and/or β (Figure 4). For example, a NLS was observed in the seventh transmembrane domain and the carboxy-terminal segment of the Ang-II receptor subtype1 (Lys-Lys-Phe-Lys-Arg) and the third intracellular loop of the apelin receptors (Arg-Lys-Arg-Arg-Arg) (Lee et al., 2004). Interestingly, a similar sequence, *i.e.* Lys-Arg-Ala-Arg-Arg, is also observed in the third intracellular loop of human and monkey UT isoforms (Figure 3) while a Lys-Gln-Thr-Arg-Arg segment is observed in rat and mouse UT. However, it is important to note that many NLS signals are still unknown and that the presence of an obvious NLS motif may mask the existence of still uncharacterized NLS sequences. Specific post-translational modifications such as palmitoylation were reported to be involved in the addressing of the receptor either to the membrane or the nuclei. For instance, it was demonstrated that de-palmitoylation of GRK6A promoted its translocation from the plasma membrane to both the cytoplasm and nucleus (Jiang et al., 2007). Such a putative palmitoylation site is also found within the seventh transmembrane domain (Cys^339^) of rat and mouse UT isoforms but is absent in the primate (human and monkey) receptor (Marchese et al., 1995; Tal et al., 1995; Ames et al., 1999). Under chemically mediated hypoxic conditions, an increase of total UT expression, was observed suggesting that hypoxia might induce *de novo* synthesis of the peptide receptor. However, a significant decrease in nuclear UT expression was reported that could be interpreted as an increase in translocation of the protein to the membrane or a decrease of internalization with concomitant nuclear translocation (Nguyen et al., 2012). Altogether, it could be noted that the subcellular UT localization could be either attributed to translocation from the cell surface and/or *de novo* synthesis (Figure 4).

### UII and URP as intracrine ligands

UII, and by extension URP, were originally thought to act as autocrine and paracrine modulators rather than as hormones (Yoshimoto et al., 2004). The term “intracrine” ligand relates to intracellular molecules binding to and activating intracellular receptors (Figure 4). Such ligands can be synthesized and targeted to the Golgi apparatus for secretion or act intracellularly either before secretion or following reuptake. The intracrine gene product might also arise from an alternative transcription initiation site, differences in mRNA maturation or translation leading to a gene product lacking secretory signals and consequently active only in the intracellular space (Figure 4) (Kiefer et al., 1994; Lee-Kirsch et al., 1999; Xu et al., 2009). To this extent, it is interesting to note that two isoforms of the human UII precursor, differing mostly by their peptide signal, were discovered (Coulouarn et al., 1998; Ames et al., 1999).

A recent study demonstrated that FITC-conjugated hUII and URP were both internalized in non-expressing UT cell lines through receptor-independent mediated endocytosis (Doan et al., 2012) (Figure 4). This receptor-independent endocytic mechanism brought a new perception of the pseudo-irreversible binding characteristics often described for the urotensinergic system. Indeed, the lack of rapid UT desensitization through classic mechanisms (acid wash or trypsin treatments) was thought to reflect a strong, pseudo-irreversible binding of the ligands (Douglas and Ohlstein, 2000). However, this pseudo-irreversible character could also be due to the ability of both endogenous peptides to reach the internal compartment of the cell. Moreover, it is yet possible that following the internalization of ligand-receptor complexes, ligands are subsequently released from internalized endosomes within the cell. As such, internalized peptide-receptor complexes can be dissociated under the acidic environment found in endosomes, giving rise to receptor recycling at the plasma membrane (Figure 4) (Giebing et al., 2005). At this point, the fate of the peptide is unknown but based on the results published by Doan et al. (2012), it is conceivable that UII, and to a lesser extend URP, could leak from the vesicle and ultimately activate intracellular receptors.

### Prospective roles of nuclear UT

The precise role of this new intracrine urotensinergic system has yet to be elucidated both in physiological and pathological conditions. However, as for other GPCRs including Ang-II and ET-1 receptors, these intracellular receptors are important regulators of physiological and pathological functions and could therefore represent new targets for therapeutic interventions (Boivin et al., 2008; Tadevosyan et al., 2012).

Elevated UII plasma levels were observed in numerous disease conditions, including hypertension, atherosclerosis, heart failure, pulmonary hypertension, diabetes, renal failure, and metabolic syndrome (Ross et al., 2010). As demonstrated, the cellular uptake of UII but not URP is increased at lower pH (Doan et al., 2012). Pathological conditions such as cancer, ischemic stroke, inflammation, and atherosclerotic plaques often induce an increase in metabolic activity and hypoxia associated with an elevated extracellular acidity (Andreev et al., 2010). In these conditions, UII would enter more easily than URP inside the cell triggering transcription of UII-associated genes by activating the nuclear receptor. Thus, the elevated concentration of UII observed during the etiology of various diseases could sustain specific cellular responses while an intracellular feedback loop could maintain a particular cellular state (Petersen et al., 2006). Interestingly, known intracrines do not present any structural or chemical similarities but are generally growth regulators that can directly or indirectly modulate angiogenic or anti-angiogenic actions. Therefore, the angiogenic actions of the urotensinergic system, reported both *in vivo* and *in vitro* (Spinazzi et al., 2006), could thus involve the activation of nuclear UT.

The urotensinergic system is also highly expressed in the central nervous system, but its physiological function is still poorly understood. UT was observed in cortical astrocytes (Castel et al., 2006), a ubiquitous type of glial cell that greatly outnumbers neurons and occupies 25% to 50% of brain volume (Bignami et al., 1991). It is noteworthy that glioblastoma multiform (GBM) is characterized by exuberant angiogenesis, a key event in tumor growth and progression and that UII, URP, and UT mRNAs were systematically found to be expressed in different glioma and glioblastoma tumors (Diallo et al., 2007). These results support a role for the urotensinergic system, and in particular nuclear UT, in human brain tumorogenesis possibly via angiogenesis regulation. Finally, in the CNS, UII is able to induce norepinephrine, dopamine, and serotonin release in noradrenergic neurons (Ono et al., 2008). Intracerebroventricular UII administration modulates cardiac homeostasis via β-adrenoreceptor activation (Hood et al., 2005). These observations bring up the idea that the presence of nuclear UT receptors could also be associated to excitatory neurotransmission. In accordance with this hypothesis, various intracrines were reported to act as neurotransmitters within the CNS (Re, 2004).

Whether specific UII or URP biological actions on the CNS and the cardiovascular system are mediated totally or in part by the nuclear UT will need further studies as well as the development of specific nuclear UT probes. Although still poorly understood, the diverse functions exerted by agonists and hormones acting on intracellular GPCRs suggest that intracrine signaling might activate cellular responses distinct from those at the cell surface for a given receptor. In the last decade, biological actions of intracrines in heart and vasculature, including those of the renin–angiotensin-system in cardiac pathology, dynorphin B in cardiac development, as well as endothelin, highlighted the importance of intracrine physiology in pathological processes such as left ventricular hypertrophy, diabetic cardiomyopathy, and arrythmogenesis. So, the presence of functional UT receptors at the cell membrane and at the nucleus will probably be a new aspect to take into account during the development of therapeutic compounds for the treatment of pathologies associated with the urotensinergic system.

## New insights into UT activation

The precise definition of the (patho) physiological roles of the urotensinergic system *in vivo* was hampered by the absence of potent and selective UT antagonists. Indeed, the lack of efficacy observed with Palosuran (ACT-058362) (Clozel et al., 2004, 2006), the only UT antagonist that reached a phase II clinical trial in patients with diabetic nephropathy, was clouded by its low antagonist potency (Behm et al., 2008). Therefore, drug discovery programs continued to focus on the identification of potent and selective UT antagonists suitable for assessment in both preclinical species and man (Maryanoff and Kinney, 2010). As reported earlier this year, Sanofi launched a phase I clinical trial regarding a long acting UT antagonist, derived from a 5,6-bisaryl-2-pyridine-carboxamide scaffold (European patent application *EP2439193*), for the treatment of diabetic nephropathy. Similarly, GlaxoSmithKline started a phase I clinical trial for the use of an UT antagonist, *i.e.* SB1440115 (United States Patent application *12,373,901*), for the treatment of asthma. Finally, over the past few years, Boehringer-Ingelheim (European patent application *EP2155748*) as well as Janssen Pharmaceutical (United States Patent application *8,193,191*) filled several patents regarding UT antagonists but no phase I clinical trial was yet reported. With the recent discovery that UII and URP could exert common as well as different biological activities (Prosser et al., 2008; Hirose et al., 2009; Jarry et al., 2010), development of selective UT antagonists has become a more complex task.

### UT as shapeshifting proteins

GPCRs represent the largest and most diverse family of cell surface receptors. These plasma membrane proteins bind their endogenous ligands in order to activate an intracellular signaling cascade that will result in a biological action. Conventional views of ligand-receptor activation considered all components of the signaling cascade to be linearly related, *i.e.* to emanate from the initial activation of the receptor. However, multiple studies pointed out the ability of some ligands to selectively trigger specific signaling pathways, therefore having collateral and not linear efficacy (Roettger et al., 1997; Kohout et al., 2004). As such, GPCRs cannot be considered as pharmacological on/off switches anymore. Their intrinsic nature rather suggests that dynamic changes in the receptor conformation, resulting from ligand binding, are a mean of information transfer (Kenakin and Miller, 2010). Hence, the propensity of GPCRs to assume multiple conformations make them allosteric proteins that are able to select specific subsets of secondary messengers depending on the ligand-induced adopted conformation. As such, various ligands were reported to possess differential functional profiles for a given receptor, as it was initially described for the CCR7 receptor (Kohout et al., 2004).

The URP sequence, strictly conserved throughout species, supports the concept that specific receptor interactions were maintained despite variation in the receptor amino acid sequence (Elshourbagy et al., 2002). Based on the specific expression of URP mRNA in several cerebral structures (rostroventrolateral medulla) and tissues (heart, seminal vesicle), it was suggested that URP rather than UII would be the biologically active peptide in the UT-associated regulation of autonomic, cardiovascular and reproductive functions (Dubessy et al., 2008). Moreover, distinct pathophysiological roles for UII and URP in hypertension have been suggested (Hirose et al., 2009). Indeed, mRNA expression of both UII and URP was up-regulated in the atrium of spontaneously hypertensive rats (SHR) when compared with age-matched Wistar Kyoto (WKY) rats. However, the specific up-regulation of URP but not UII mRNA in aorta and kidney of SHR rats supported the idea that these peptides might act individually in various biological systems (Hirose et al., 2009). Accordingly, it was demonstrated that UII and URP were able to exert not only common but also divergent physiological actions clearly suggesting the propensity of these two endogenous ligands to select a specific UT conformation (Prosser et al., 2008; Jarry et al., 2010; Doan et al., 2012). The concept of biased agonism has recently emerged from various studies, putting forward the notion that specific ligand-induced conformational changes can lead to particular signaling (Patel et al., 2010). In isolated ischaemic heart experiments, UII and URP were both able to reduce myocardial damages through creatine kinase reduction but only UII was able to reduce atrial natriuretic peptide (ANP) production (Prosser et al., 2008). Supporting the idea that UII and URP interact with UT in a distinct manner, it was recently demonstrated that URP, an equipotent UII paralog, was able to accelerate the dissociation rate of membrane bound ^125^I-hUII while hUII had no noticeable effect on URP dissociation kinetics (Chatenet et al., 2012a). Altogether, these results suggest that each ligand is able to select a specific UT conformation that triggers definite biological activities for each of these two peptides.

The peptide N-terminal region was initially pointed out as potentially involved in the observed biological activity differences between UII and URP (Prosser et al., 2008). However, it is only recently that this region was clearly associated with a putative differential binding mode of hUII and URP (Chatenet et al., 2012a). Using exocyclic Ala-derivatives of hUII, acting as very potent ligands of the UT receptor (Brkovic et al., 2003), dissociation kinetics experiments revealed a putative interaction between UT and the glutamic residue at position 1 of hUII. Indeed, it was observed that the replacement of this residue by an alanine moiety, *i.e.* [Ala^1^]hUII, provoked an increase of the dissociation rate of hUII but not URP (Chatenet et al., 2012a). In agreement with this view, an important electrostatic interaction between Glu^1^ of hUII and its receptor was previously reported in docking studies (Lescot et al., 2008). No other substitution was able to induce such pharmacological changes. Because this compound was reported to exert almost equipotent contractile activity compared to hUII and URP, the lost of a putative specific interaction with UT was thought to have generated an analog behaving as an URP derivative therefore acting at the URP-associated orthosteric binding site. Supporting this hypothesis, hUII(4–11), considered as the minimal hUII fragment to exert full biological activity, was also able to alter hUII but not URP dissociation rate (Chatenet et al., 2012a). This N-terminal segment could thus be crucial for signaling pathway selection upon activation and its deletion could lead to signaling mechanism misinterpretation, all UII truncated analogs potentially acting as URP derivatives.

**Figure 6:**
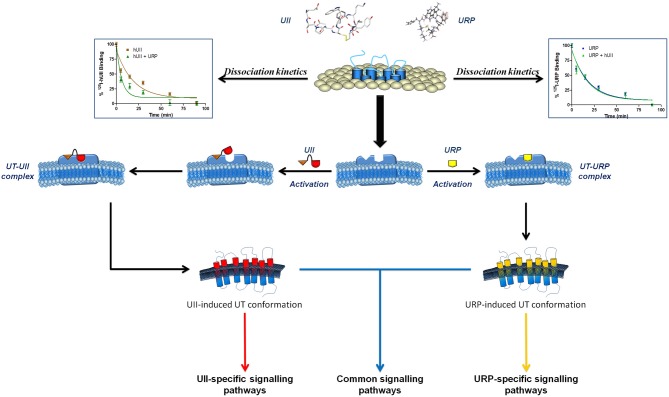
**Schematic representation of a proposed binding mode for UII and URP.** Following the initial interaction between the N-terminal domain of UII and its receptor, UT undergoes a conformational change aimed at welcoming the C-terminal domain of UII, characterized by an intracyclic β-turn, in a specific binding pocket. However, URP, lacking this N-terminal portion and characterized by the presence of an intracyclic γ-turn, is also able to bind UT but in a slightly different manner, probably characterized by the activation of a different subset of signaling pathways. Modified from Chatenet et al. (2012a).

Overall, these results support the presence of specific pockets/interactions within UT, aimed at selecting distinct UT conformations that can differentiate UII and URP biological activities (Figure 6). Briefly, it is hypothesized that upon the initial UII-UT interaction, involving the N-terminal region of UII, UT undergoes conformational changes aimed at welcoming the C-terminal domain of UII, characterized by an intracyclic β-turn (Carotenuto et al., 2004). To the opposite, URP, lacking this N-terminal portion and characterized by the presence of an intracyclic γ-turn (Chatenet et al., 2004), would bind UT in a slightly different manner; ultimately triggering a slightly different subset of signaling pathways. These observations have clearly highlighted the crucial need to reassess the development of pan UT antagonists, *i.e.* blocking UII- and URP-mediated receptor activation, and to develop new analogs of the urotensinergic system aimed at discriminating UII- or URP-mediated biological activities. Such compounds would allow a better understanding of the pathophysiological roles of the urotensinergic system and also expand our knowledge on allosteric modulation of class A GPCRs.

#### Allosteric modulation of the urotensinergic system

As demonstrated, the urotensinergic system is far more complex than previously thought with the presence of nuclear receptors and UII/URP specific as well as common actions. For the past two decades, identification of peptidic and non-peptidic agonists and antagonists of the urotensinergic system has gathered much interest for the treatment of various cardiovascular pathologies. An extensive review regarding the various peptidic and non-peptidic ligands of the urotensinergic system, all acting as competitive compounds, is beyond the scope of this review but more details can be found elsewhere (Maryanoff and Kinney, 2010).

Additional biological complexity, but also novel opportunities for drug discovery, has arisen from the fact that many GPCRs possess allosteric binding sites (Christopoulos and Kenakin, 2002). Similar to the initial concept of agonism, *i.e.* linear efficacy, antagonism has been historically viewed as a simple “turning off” of the receptor. As such, this non-accommodating mechanism does not allow any agonist to impart information to the receptor, the orthosteric binding site being occupied by the competitive antagonist. However, an allosteric modulator binds to its own site, different from the orthosteric site, forming a complex characterized by the concomitant presence of the endogenous agonist and the allosteric modulator. Such modulators can alter the biological properties of the endogenous orthosteric ligand either via changing its affinity, its efficacy, or both (Leach et al., 2007; May et al., 2007). This type of antagonism, termed permissive, can modify the reactivity of the receptor toward the agonist probably through conformation selection and stabilization of one or part of the receptor states. Targeting receptor allosteric sites can offer the possibility of greater selectivity due to a lower sequence conservation within allosteric pockets across subtypes of a given GPCR, as well as the potential to fine-tune physiological signaling in a more spatial and temporally-selective manner (Kenakin, 2011).

**Figure 7:**
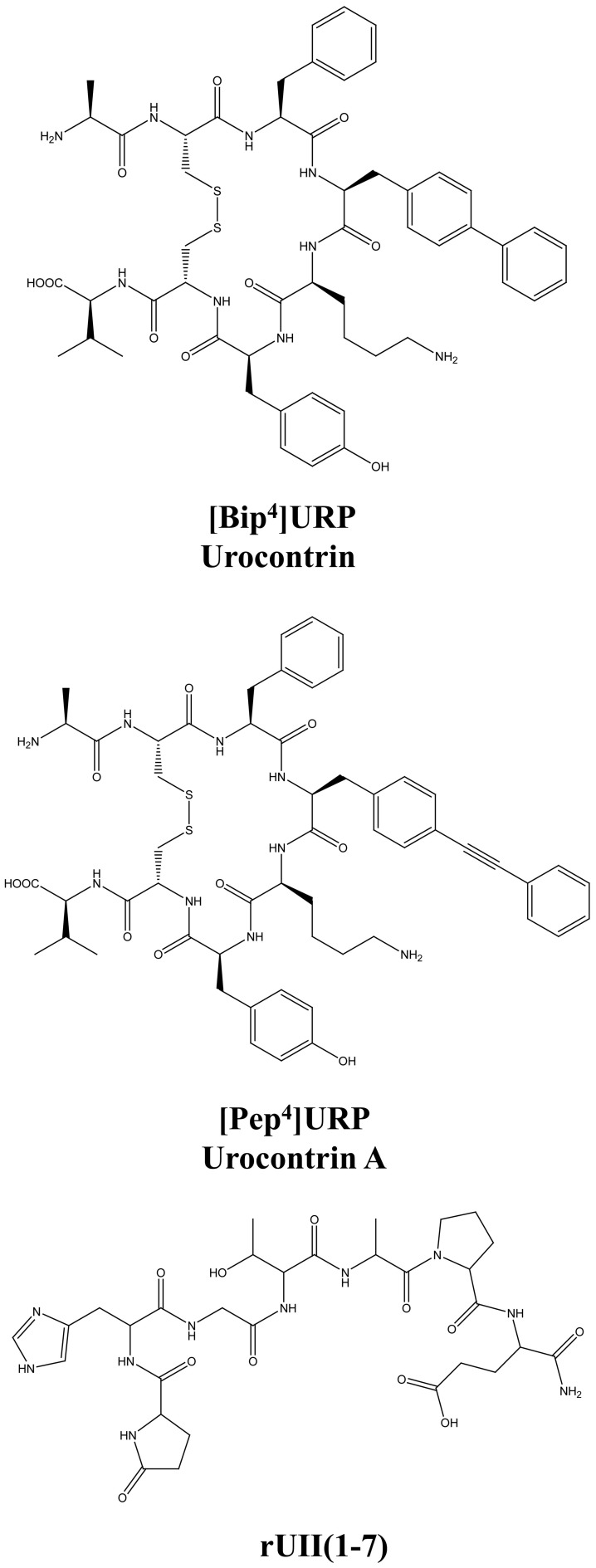
**Structure of the novel UT allosteric modulators of the urotensinergic system, *i.e.* [Bip^4^]URP (urocontrin), [Pep^4^]URP (urocontrin A; UCA), and rUII(1–7).** Modified from Chatenet et al. (2012a,b).

**Figure 8:**
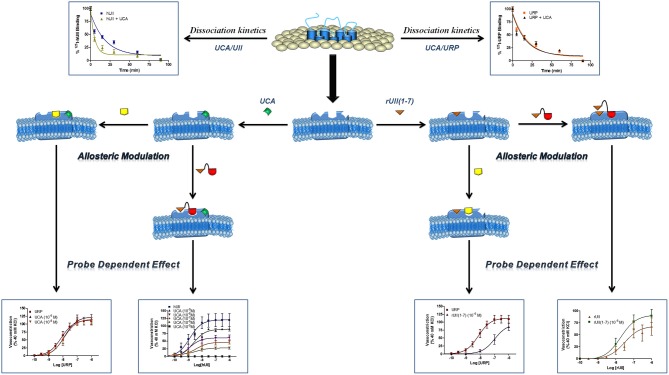
**Schematic representation of a proposed allosteric modulation of the urotensinergic system by urocontrin A and rUII(1–7).** By acting at an allosteric binding site, UCA is able to modify the receptor topography preventing the proper interaction of UT with the linear UII N-terminal region ultimately leading to an inefficient activation characterized by a reduced efficacy. On the opposite, such a receptor conformational change has no effect on URP-mediated action. Conversely, binding of the rUII(1–7) N-terminal segment, initiates a topographical change that antagonizes the effect of URP, but not UII. Modified from Chatenet et al. (2012a).

The urotensinergic system, encompassing two endogenous peptides, provides potential for allosteric compounds to differentially modulate individual peptide responses, a behavior termed “probe dependence” (Kenakin, 2008). During the course of structure-activity relationship studies on URP derivatives, two compounds, *i.e.* [Bip^4^]URP and [Pep^4^]URP, termed urocontrin and urocontrin A (UCA) respectively, showed a specific behavior that has set them apart from known UT antagonists (Figure 7) (Chatenet et al., 2012a,b). Indeed, these compounds were able to selectively and significantly reduce hUII-induced contraction without altering URP-mediated vasoconstriction (Chatenet et al., 2012a,b). For instance, the efficacy of hUII-induced rat aortic ring vasoconstriction was significantly reduced (~31%) by a pre-treatment with a nanomolar concentration of UCA (Figure 8). Interestingly, this ability to selectively and significantly reduce UII-induced contraction was not specie-dependent since a similar effect was observed on cynomolgus monkey aortic rings. To the best of our knowledge, only two other UT ligands exerted insurmountable activity (Herold et al., 2003; Behm et al., 2010). However, none of them could differentially alter hUII and URP biological activity. The insurmountable nature of urocontrin and UCA antagonism was attributed to an allosteric modulation of UT. Indeed, an excess of UCA accelerated the ^125^I-hUII dissociation rate, thus suggesting that the binding of the antagonist changes the receptor conformation in such a way that the radioligand is released from the receptor. Accordingly, no difference in ^125^I-URP dissociation kinetics was observed in similar conditions (Figure 8). The apparent absence of effect on the URP pharmacological profile by UCA was attributed to its ability to select a receptor conformation through functional allosteric modulation that impairs hUII-associated actions but not URP-mediated biological activities.

As stated above, for a given receptor, an allosteric modulation that depends on the type of orthosteric ligand used is referred to as “probe dependence” (Kenakin, 2005; Keov et al., 2011). This probe dependence phenomenon supports the idea that the two endogenous ligands, despite depicting a high structure homology and recognizing a similar binding pocket, represent chemically distinct entities interacting in different structural environments within the orthosteric pocket. Because hUII and URP differ only by the length and composition of their N-terminal domain (Vaudry et al., 2010), it was postulated that this region could be involved in their putative different binding modes. Corroborating this hypothesis, the hUII counterpart of UCA, *i.e.* [Pep^7^]hUII, acted as a weak but full agonist of the UT receptor (Chatenet et al., 2012a). This N-terminal domain is thus able to modulate the topology of the receptor in such a manner that the C-terminal domain of UII is able to trigger receptor activation. However, could this N-terminal segment be biologically active? As an agonist, the N-terminal domain of rat UII [rUII(1–7)], *i.e.* Pyr-His-Gly-Thr-Ala-Pro-Glu-amide (Figure 7), was unable to induce the contraction of rat aortic rings (Chatenet et al., 2012a). Amazingly, pre-treatment of rat aortic rings with rUII(1–7) induced an apparent increase in rUII contractile efficacy while reducing the potency and the efficacy of URP-mediated vasoconstriction (Chatenet et al., 2012a). These results clearly suggested that rUII(1–7) acted as a probe dependant allosteric modulator on rUII- and URP-mediated vasoconstriction (Figure 8) (Chatenet et al., 2012a). Since all UII isoforms possess different N-terminal domains, it is hypothesized that these regions could act as specie-selective specific URP modulators but there is currently no clue regarding an endogenous production of those N-terminal UII domains *in vivo*.

## Conclusions

These latest findings about the urotensinergic system will probably generate a considerable interest within the scientific community. First, the discovery of UT on the nuclear membrane and the presence of intracellular ligands open up new avenues in UT signaling physiology. In general, nuclear-localized receptors may regulate distinct signaling pathways, suggesting that biological responses mediated by GPCRs are not only initiated at the cell surface but might result from the integration of extracellular and intracellular signaling pathways. These receptors are therefore well-positioned to play major roles in the physiological and pathophysiological responses associated with their endogenous ligands. Finally, the discovery of allosteric modulators of the urotensinergic systems such as urocontrin, UCA, and rUII(1–7), will surely enable a better understanding of the urotensinergic system by allowing to discriminate *in vitro* and *in vivo* specific biological actions mediated by UII and/or URP. Therefore, these unique derivatives will be useful as chemical templates for the rational design of novel UT receptor ligands, as well as pharmacological tools for *in vitro* and particularly *in vivo* studies aimed at clarifying the role(s) played by the UII/URP/UT receptor system in physiology and pathology.

### Conflict of interest statement

The authors declare that the research was conducted in the absence of any commercial or financial relationships that could be construed as a potential conflict of interest.
